# High prevalence of syphilis among demobilized child soldiers in Eastern Congo: a cross-sectional study

**DOI:** 10.1186/1752-1505-5-16

**Published:** 2011-09-06

**Authors:** Raphael Kabangwa Senga, Prosper Mukobelwa Lutala

**Affiliations:** 1 Appui Médical Intégré Aux Activités de Laboratoire (AMI-LABO), 68 Golf Avenue, Goma, Boite postale 187, Congo and Département des Sciences de base, Université de Goma, 2 Avenue Himbi, Goma, Boite postale 204, Congo; 2 United Nations Volunteers, Central-East Zone, Health éducation Unit Compound, Lilongwe, P.O. Box 30135, Malawi and Département de Médicine de Famille, Université de Goma, 2 Avenue Himbi, Goma, Boite postale 204, Congo

## Abstract

**Background:**

Syphilis, a known major public health issue for soldiers during periods of conflict, is exacerbated in the Democratic Republic of Congo due to widespread sexual violence. However, there has been no previous study to determine the extent of this problem. Therefore, we determined the prevalence of syphilis among young demobilized soldiers.

**Methods:**

Screening of syphilis using the rapid plasma reagin test and the *Treponema pallidum *hemagglutination assay was conducted in three transit sites of soldier reintegration in 2005. The Fisher Exact probability test was used to compare results.

**Results:**

The prevalence of syphilis was found to be 3.4%, with almost equal distribution in respect to sex, location.

**Conclusion:**

Syphilis continues to be highly prevalent in demobilized child soldiers in Eastern Congo. Syphilis screening tests are recommended.

## Background

Syphilis and to some extent other sexually transmitted infections (STIs) are a major public health issue for soldiers during periods of conflict. In the Democratic Republic of Congo (Congo), these have been exacerbated by widespread sexual violence. Child soldiers are particularly vulnerable due to several factors: incomplete maturation, low social conditions, use as sexual workers by superiors, and their promiscuous environment. During World Wars I and II and subsequent armed conflicts throughout the world, syphilis has played an unprecedented role in soldier morbidity [1]. The Congo, with almost two decades of armed conflicts, is characterized by widespread sexual violence [2,3]. In the Congo and other African countries, recruitment of child soldiers has been largely practiced despite its war-crime characterization as defined by the Rome Statute of the International Criminal Court [4].

As a war nears its end, disarmament, demobilization, and reinsertion (DDR) of combatants is a compulsory post-conflict step. In the Congo, all child soldiers undergo this process, which allows soldiers who desire, or who are children, to return to civilian life. At the transit camp soldiers undergo compulsory syphilis testing. To prevent possible spread of the disease upon reintegration, those who test positive undergo treatment.

Despite a number of studies dealing with syphilis prevalence in various contexts, to our knowledge, little is known about syphilis prevalence in demobilized soldiers, and particularly in child soldiers. Therefore, we determined the prevalence of syphilis among this group in the Congo.

## Methods

### Design population and sampling

This cross-sectional study was conducted in three Transit and Reception Centers during DDR in Goma, Congo, between April 14 and June 14, 2005. Study subjects were young (extremes: 10 and 20 years old), newly-demobilized soldiers who, after spending some period in army/rebellion militias, were going through preparation for community integration. Some participants were also receiving vocational training. All participants were recruited by the national army or rebellion militia when they were less than 15 years old. Three hundred participants were contacted, and 163 volunteered to undergo screening for syphilis.

### Data collection

After written informed consent was obtained from each participant and each participant underwent appropriate pretest counseling, venous blood was collected and transferred to the laboratory. Following identification and verification of the samples for conformity, the blood cells were centrifuged and sera were separated from cells immediately after clotting. Syphilis serostatus was determined by the rapid plasma reagin (Lampole Laboratories, Princeton, NJ., USA) test and the *Treponema pallidum *hemagglutination assay (Serodia1-TPHA; Fujirebio Inc., Tokyo, Japan), according to the manufacturers' instructions. Remaining sera were stored at -20°C. Results were considered positive if both tests were found to be reactive. Posttest counseling was provided by trained counselors irrespective of the results.

### Statistical analyses

Descriptive statistics were generated by using the subjects' demographic characteristics, and results were presented as percentages. Data and associations between demographic characteristics and syphilis tests were analyzed using Fisher's Exact Test [5].

### Ethical considerations

Ethical clearance to conduct the study was granted by the ethical review board of the University de Goma. Written informed consent for publication was sought and obtained from each patient or a relative before the sample of blood was collected.

## Results

### Socio-demographic characteristics

The sociodemographic characteristics of the study are presented in Table 1

**Table 1:** Socio-demographic data

Variables	Frequency	Percent
**Sex of the participants**		
Males	244	89.7
Females	28	10.3
**Age range (years)**		
10-15	67	25.5
15-18	165	62.7
> 18	26	9.9
Missing values	5	1.9
**Participant location**		
Karibu CAJED* Transit and Reception Center	134	51.0
SOS Grand-Lacs Transit and Reception Center	69	26.2
Divas Transit and Reception Center	60	22.8

**Total**	**263**	**100**

*Concert d'Actions pour Jeunes et Enfants Défavorisés

A total of 263 participants, who were mostly males (244/263; 89.7%), between the age of 15 and 18 years old (62.7%), and from the Karibu C Transit and Reception Center (51%), were recruited. (Table 1)

### Prevalence of syphilis

Table 2 shows the prevalence of syphilis and its comparison across variables. The prevalence of syphilis was 3.4%. The distribution of syphilis serology results did not show any difference in terms of residence location (*p *= 0.9049), sex of participants (*p *= 0.2341), but there was a difference between the age ranges (*p *< 0.0001) (Table 2).

**Table 2:** Prevalence of syphilis

	Positive	Negative	p- (Fisher 2-tails)
**Participant's Locations**			
CAJED Karibu Transit and Reception Center	4(3.0)	130(97.0)	
			0.9040
Grand-Laces Transit and Reception Center	3(4.3)	66(96.7)	
Divas Transit and Reception Center	2(3.3)	58(96.7)	
**Sex of participants**			
Males	7(2.9)	237(97.1)	0.2341
Females	2(7.1)	26(92.9)	
**Ages ranges **(in years)			
10-15	0(0.0)	67(100)	
15-18	8(4.8)	157(95.2)	< 0.0001
> 18	1(3.8)	25(96.2)	
Missing values	5(100)	0(0.0)	

Total	9(3.4)	254(96.5)	

## Discussion

The prevalence of syphilis among young demobilized soldiers was found to be quite high at 3.4% in Eastern Congo (Table 2). The results can be interpreted that child soldiers are at disproportionately higher risk of experiencing sexual violence at an early age.

While lower than the prevalence reported among soldiers in Ethiopia (16.7%) [6], this prevalence was higher than that found among pregnant women in Kinshasa [7], and similar to victims of sexual violence in nearby South-Kivu province [8]. Reproductive health assessments of internally displaced women residing in camps, and counterparts residing in surrounding host communities, showed the syphilis prevalence to be 4 and 0.5%, respectively. This may be a proxy of high syphilis prevalence in soldiers who reside nearby. Contrary to our results, surprisingly in a similar war-torn setting in Ibadan, Iran, syphilis prevalence was lower (0.1%, with genital ulcer prevalence of 1.9%) [9]. Reasons are still unclear.

The higher prevalence in females, and in 16-18 year-olds soldiers (Table 2), needs cautious interpretation, because the number of girls is too low and sexual activity is intense at this age range. Furthermore, among people of a young age with limited sexual experience, the risk of STIs is high.

### Limitations

This study must be interpreted in light of several limitations. Our investigations examined one STI, while groups in conflict settings are prone to a range of STIs. The role of ulcerative STIs in transmission of HIV could justify the current study. The nonrandom selection of our sample may not allow any generalization of our results. Some social/behavioral determinants that have a bearing in syphilis (and STI) acquisition, such as condom use, sexual partner numbers, sexual intercourse type, and duty duration were beyond the scope of the current study and were not investigated. Lack of quality control could jeopardize the validity of the results.

Nonetheless, the association of rapid plasma reagin-*Treponema pallidum *hemagglutination assay remains appropriate in the diagnosis of syphilis in our challenging work conditions in the Congo [10]. The World Health Organization advocated STI screening to control sexual transmitted infections in recent years, using simple rapid points of care targeting mainly high-risk groups in the community, such as military recruits and company employees [11], adolescents, and sex workers [12]. Early detection of symptomatic and asymptomatic infections is a key element in the public health package for STI control [13]. A recent meta-analysis of prenatal screening programs based on studies conducted in the United States and other countries found that low- and middle-income populations showed a reduction in the incidence of perinatal deaths and congenital syphilis in live-born infants after appropriate treatment [14].

## Conclusion

The prevalence of syphilis in demobilized child soldiers was high in Goma, especially in 16- to 18-year-olds and in females. Efforts should be taken to generalize such screening to other demobilization sites and extend testing to other STIs, including HIV. A study including all sexual behavioral factors as well as the determinants of syphilis (and other STIs) in demobilized child soldiers is warranted in the near future.

## List of abbreviations used

AMI-LABO: Appui Médical Intégré aux activités de laboratoire; CAJED: Concert d'Actions pour Jeunes et Enfants Défavorisés; DDR: Disarmament, demobilization, and reinsertion; DOCS: Doctors on Call for Service; HIV: Human immunodeficiency virus; STI: Sexually transmitted infection; TPHA: *Treponema Pallidum *haemagglutination Assay; UN Volunteers: United Nations volunteers; UNDP: United Nations Development Programme; UNICEF: United Nations Children's Fund; USA: United States of America.

## Competing interests

The first author is one of the study's funders and the director of the Ami-Labo (the laboratory that carried out the testing of all blood samples). However, the laboratory did not play any role in the conception, data collection, analysis, or reporting of the current research.

## Authors' contributions

RKS conceived the idea, collected the data, and gave input in the manuscript drafting. PML designed the study, analyzed and interpreted the data, and drafted the manuscript. All authors read and approved the final manuscript.

## Authors' details

RKS: Graduate clinical biologist, lecturer at Département des sciences de base the school of Medicine (University of Goma), and director of the AMI-Labo, the provincial referral laboratory for the North Kivu province; Goma Congo/DRC.

PML: HIV Zonal supervisor/MOH Malawi and family physician Département de Médecine de Famille, Université de Goma.
